# Dissemination of validated health literacy videos: A tailored approach

**DOI:** 10.1002/cam4.4572

**Published:** 2022-02-02

**Authors:** Shannon M. Blee, Jamil Facdol, Margie D. Dixon, Viraj Master, Jeffrey M. Switchenko, Rebecca D. Pentz

**Affiliations:** ^1^ Emory University Winship Cancer Institute Atlanta Georgia USA; ^2^ Grady Memorial Hospital Atlanta Georgia USA; ^3^ Emory University School of Medicine Atlanta Georgia USA; ^4^ Emory University Rollins School of Public Health Atlanta Georgia USA

**Keywords:** educational tools, health literacy, implementation science, dissemination, educational videos

## Abstract

**Background:**

Previously, we showed that chemotherapy terminology is difficult for patients to understand. Therefore, we developed short videos explaining key terminology and though proven effective, they will only be helpful if appropriately disseminated. Therefore, we aimed to determine the best dissemination method at three different healthcare settings.

**Methods:**

With consent, we interviewed healthcare workers from (1) an academic cancer center, Winship Cancer Institute (Winship) serving higher SES patients, (2) an inner‐city, safety‐net hospital Grady Memorial (GMH), (3) clinics serving rural Georgia, from the Winship Community Network (Network). All interviews were transcribed and analyzed using a semantic content analysis method. Suggested dissemination plans were then implemented.

**Results:**

Twenty‐two Winship, 11 GMH, and 4 Network healthcare workers were interviewed. Seventy‐two percent (*n* = 8) of the GMH and 100% (*n* = 4) of Network healthcare workers felt that the best place for patients to view the videos was in the clinic, compared to 27% (*n* = 6) of the Winship clinicians. 68% (*n* = 15) of the Winship clinicians stated an app would be the most useful format, compared to 27% (*n* = 3) at GMH, and 0% at Network sites. Video viewing increased after dissemination plans were implemented.

**CONCLUSION:**

Educational materials explaining oncology treatment terminology enhance patient understanding, yet without proper dissemination, these tools may never reach the intended patient population. Our study shows that dissemination plans need to be tailored to each individual patient population, with rural and lower SES patients needing to view the videos during clinic visits, and patients of more means viewing them using technology at home.

## Graphical abstract

Dissemination plans of validated health literacy videos explaining chemotherapy terminology need to be tailored to each individual healthcare setting to ensure they benefit the intended patient population.

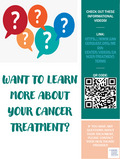

## 1 INTRODUCTION

Low health literacy and patient misunderstanding of terminology is a critical issue in oncology^1^, ^2^ and it can impact the adequacy of informed consent, adherence to treatment^3^, ^4^, and patient outcomes.^5^, ^6^ In order to improve patients’ health literacy and understanding of terminology, many educational tools, specifically those using technology, have been developed and have been proven successful, allowing patients to make more informed decisions about their cancer treatment.^7^, ^8^, ^9^ In particular, video‐based interventions have been shown to be effective in a multitude of settings.^10^, ^11^, ^12^, ^13^, ^14^ Previously, and concurrent with the results of numerous studies, we have shown that chemotherapy terminology is challenging for patients to understand.^1^, ^2^, ^15^ Since videos have been proven successful at increasing patients’ levels of understanding of complex terminology, we developed short, animated videos to explain key terminology of chemotherapy treatment. After testing these videos in both an urban and rural population, the results indicated that they significantly improved patient understanding.^15^, ^16^ However, although the videos are freely downloadable on CancerQuest (https://www.cancerquest.org/media‐center/videos/cancer‐treatment‐terms), they will be more helpful if appropriately disseminated.

Although the success of educational tools at improving health literacy is well‐documented, dissemination of educational tools is not as well‐documented, and often educational tools are not incorporated in a manner that encourages patient use. In 1999, Richard et al. emphasized the importance of pairing “evidence‐based medicine” with “evidence‐based implementation,” yet a recent review found that a limited number of educational initiatives in oncology had any implementation measures.^17^, ^18^ In another study, even after a video‐based educational tool for prostate cancer was proven effective for improving patient comprehension, no dissemination plan was ever implemented due to copyright issues.^10^ For the educational tools that have been implemented, very limited research exists on the success of the implementation measures used. While educational tools may be effective at improving patient’s health literacy, they will not be as beneficial to patients if they are not properly disseminated in a manner that best suits patients’ needs.

Since past research has shown that it is challenging to properly implement educational tools, the purpose of this study was to interview healthcare workers at three different healthcare settings with disparate populations—a higher SES hospital, an underserved, inner‐city hospital, and community clinics that serve rural populations—about the best dissemination methods for their site and then to implement and track the success of the recommended dissemination methods. Our overall objective was to propel these validated health literacy videos from the academic community to patients.

## 2 METHODS

This study was approved by the Emory Institutional Review Board and all participants consented to participate.

### 2.1 Recruitment

Participants were recruited for this study from three different healthcare sites: (1) Winship Cancer Institute (Winship); (2) Grady Memorial Hospital (GMH); (3) Winship Network sites (Network). These three different sites were chosen because each site serves a distinct cancer patient population. Winship’s main clinic is located in an urban center and its patients are predominantly higher social economic status (SES), that is employed and insured. Only 6% (2257/37031) of Winship patients seen in 2020–2021 were unemployed. In comparison, Grady is a public, inner‐city hospital that serves a lower SES patient population with high numbers of patients being unemployed and underinsured. Among the 50 Grady patients we interviewed to establish the efficacy of the educational videos, only 8 were employed either full‐time or part‐time (16%).^15^ The third healthcare site interviewed included three different Winship Network sites that serve a predominately rural population. In order to determine rurality, we used the United States Department of Agriculture’s 2013 Rural‐Urban Continuum Codes (RUCC)^19^, which distinguishes metropolitan counties (RUCC 1‐3) from non‐metro counties (RUCC 4‐9). Each of the three Winship Network sites are in counties categorized as RUCC 3 (Counties in metro areas of fewer than 250,000 population) but each border on RUCC 6 counties (Nonmetro—Urban population of 2500 to 19,999, adjacent to a metro area) and RUCC 8 counties (Nonmetro—Completely rural or less than 2500 urban population, adjacent to a metro area). Our previous study testing the videos at these three sites easily accrued only rural patients (those living in RUCC 4‐9 counties).^16^ As is well known, rural populations have higher poverty rates and fewer high school graduates, which is true of rural Georgia.^20^

Eligible clinicians at Winship included both those treating solid tumors and hematological disorders. Winship clinicians were recruited by obtaining a list serve from Winship of all clinicians. At GMH, we conferred with the Grady Oncology Working Group to identify the providers to interview and were advised that we should expand our interview pool beyond physicians to include research coordinators, administrators, and other staff since they participated in patient education. We, therefore, recruited a more diverse set of providers using a list of potential participants provided by the Grady Oncology Working Group. Any healthcare worker who participated in the Winship Network was eligible, and the directors of the Winship Cancer Network identified participants. All recruited participants were sent an email with an introduction to the interview study and an information sheet, followed by an additional two emails if they did not respond to the initial email. To enhance enrollment, the study was introduced at working group meetings as well. Upon an email response, a research assistant set up a time to conduct the interview via Zoom or phone call. The research assistant also sent the participant a link to view the videos before the scheduled interview. The link consisted of twenty videos describing chemotherapy terminology, with each approximately one‐minute in length. Our goal was to interview sufficient numbers of healthcare workers at each site so that using qualitative analysis methodology, we obtained saturation of themes, with no new methods of dissemination being mentioned at each site. Saturation of themes can be accomplished with as few as 6 interviews but it is typically reached around 20 interviews.^21^

### 2.2 Interviews

The interview was developed based upon a literature review and was reviewed by an expert Intervention Development, Dissemination, and Implementation (IDDI) team. The draft was then cognitively tested with physicians and staff who were not oncologists and then finalized.

The questions posed about the educational videos to the healthcare worker included: when patients should view them, the best place for patients to view them, how to incorporate them into clinic workflow, who would introduce them, what format would be most useful, what problems or barriers there might be in sharing them, if they are appropriate for their patient population, if they should be translated to a different language, and if it would be helpful to encourage the entire family to view them.

#### 2.2.1 Procedures

Verbal consent was obtained to proceed with the interview and completion of the interview‐documented consent. Before beginning the interview, the participant had an additional opportunity to view the videos in order to ensure the participant was familiar with the videos. All participants were asked a question, and participants could provide multiple answers for each question posed. Each answer given was included in the qualitative analysis. An interview methodology was used, rather than a questionnaire, for two reasons: (1) it allowed the interviewee to suggest any dissemination method that may benefit their patient population, rather than picking from a predetermined list and (2) the interviewer could probe to gain additional clarification from participants and allow them to expand on their answers.^22^

#### 2.2.2 Qualitative analysis

The interviews were audio‐recorded, transcribed, and qualitatively analyzed using a semantic content analysis method to systemically extract meaning from the transcribed interviews.^23^ Code books were created by two independent investigators, and the final code book was approved by the PI. All interviews were double coded using the final code book. Discrepancies were resolved by consensus, and continued disagreements were reviewed by a tie‐breaking, third independent coder. The frequency of mention of each theme, in this case, the frequency of mention of each method of dissemination, was computed.

### 2.3 Dissemination

Once the interviews were qualitatively analyzed and the frequencies of mentions of each dissemination method were calculated, the ethics team worked with key administrators and publicity personnel at each site to disseminate the videos utilizing the methods most frequently mentioned by that site’s interviewees. The ethics team, consisting of a research ethicist, a senior research coordinator, and two research assistants, had monthly update meetings with the team at Winship and GMH. Using both the CancerQuest analytics and social media views, preliminary results from the dissemination methods implemented were recorded. The ethics team also worked with the nurse educators at the Network sites.

#### 2.3.1 Quantitative analysis

*p*‐values were obtained using Fisher’s exact tests to compare healthcare workers’ opinions on how to disseminate the videos at the three clinic sites. Analysis was performed using SAS 9.4 (SAS Institute Inc.), and statistical significance was assessed at the 0.05 level.

## 3 RESULTS

Hundred and eight participants were approached about participation in this study and 37 healthcare workers consented to participate (33.9%). Fifty‐nine healthcare workers did not respond to the recruitment email, 3 healthcare workers did not login in for the interview and did not respond to follow‐up emails to re‐schedule the interview, and 9 were excluded from the study because they did not see patients or coordinate patient education. Of the participants approached at each site, Winship had a response rate of 28% (22/78), Grady had a response rate of 68% (11/16), and Network sites had a response rate of 28% (4/14). The median age of the 37 participants was 42 years old; 19 were male (51.35%); 18 were Caucasian/white (48.65%), and 29 were physicians (78.38%). The demographics of all participants are shown in Table 1. Saturation of themes was achieved at both the Winship and Grady sites, but not at the Network site.

**TABLE 1:** Healthcare worker demographics

Characteristic	Winship Cancer Institute (*N* = 22)	Grady Memorial Hospital (*N* = 11)	Winship network sites (*N* = 4)
**Age**			
25–39	11 (50%)	3 (27.3%)	1 (25%)
40–49	5 (22.7%)	5 (45.5%)	3 (75%)
50–59	3 (13.6%)	1 (9.1%)	—
60–79	2 (9.1%)	1 (9.1%)	—
Prefer not to answer	1 (4.5%)	1 (9.1%)	—
**Gender**			
Male	14 (63.6%)	3 (27.3%)	2 (50%)
Female	8(36.4%)	8 (72.7%)	2 (50%)
**Ethnicity**			
White	13 (59.1 %)	1 (90.1%)	4 (100%)
Asian or Pacific Islander	3 (13.6%)	3 (27.2%)	—
Black or African American	1 (4.5%)	5 (45.5%)	—
Spanish origin or descent	1 (4.5%)	2 (18.2%)	—
Mediterranean	1 (4.5%)	—	—
Other	3 (13.6%)	—	—
**Job description**			
Physician	21 (95.5%)	5 (45.5%)	3 (75%)
Administrator	—	2 (18.2%)	1 (25%)
Registered nurse	—	2 (18.2%)	—
Research coordinator	—	1 (9.1%)	—
Pharmacist	1 (4.5%)	—	—
Social worker	—	1 (9.1%)	—
**Specialty of physicians**			
Hematology oncologist	8 (36.4%)	3 (27.3%)	—
Breast	2 (9.1%)	2 (18.2%)	—
GI	4 (18.2%)	—	—
GU	3 (13.6%)	—	—
Community oncologist	—	—	3 (75%)
Head and neck	2 (9.1%)	—	—
Aerodigestive	2 (9.1%)	—	—
**Years practicing post fellowship**			
Current fellow	—	1 (9.1%)	—
0–5 years	9 (40.9%)	2 (18.2%)	1 (25%)
6–10 years	4 (18.2%)	2 (18.2%)	2 (50%)
11–15 years	5 (22.7%)	—	—
16–25 years	3 (13.6%)	—	1 (25%)
More than 25 years	1 (4.5%)	—	—

### 3.1 Interview results

#### 3.1.1 Winship Cancer Institute

Twenty‐two clinicians at Winship Cancer Institute participated in the study. While some clinicians felt the videos should be in the clinic (6/22; 27.2%), the majority stated the videos should be viewed at home either before consent (10/22; 45.5%), or after consent (8/22; 36.4%). The preferred methods of making the videos available were sending the link through the patient portal (9/22; 40.9%), providing a handout with a link to CancerQuest (6/22; 27.2%), or other methods, such as creating an interactive graphic novel, or inserting the link into the patient’s online reading material (6/22; 27.2%). If they were to be incorporated into the clinic, clinicians stated tablets, or iPads would be best (10/22; 45.5%). The majority of clinicians stated a nurse would be best to introduce the videos (15/22; 68.2%), that an app would be the best format to use (15/22; 68.2%), that the videos should be translated to Spanish (14/22; 63.6%), and that they should be shared with the patient’s caregivers (16/22; 72.7%). While most of the clinicians felt that the videos were appropriate for their entire patient population (14/22; 63.6%), some felt they were too basic (5/22; 22.7%). Thirty‐one percent (7/22) of Winship clinicians thought accessing the information or the technology needed to view the videos could pose a barrier in sharing the videos; however, despite this concern six of these clinicians still felt that an app was the best format to share the videos, but suggested that multiple points of access may be beneficial. Twenty‐two percent (5/22) of clinicians felt there were no barriers.

#### 3.1.2 Grady Memorial Hospital

Eleven healthcare workers at Grady Memorial Hospital participated in the study. The majority of healthcare workers felt the videos should be viewed before consent (8/11; 72%), or during consent (2/11; 18%). Different from Winship, most of the healthcare workers thought the best place for patients to view the videos was in the clinic (8/11; 72%), using the available computer screens/monitors in the patient rooms (6/11; 54%), or tablets if they could be made available (5/11; 45%). Healthcare workers agreed that a nurse would be the best person to introduce the videos to the patients (9/11; 81.8%), that the videos were appropriate for their entire patient population (9/11; 81.8%), that they should be translated to Spanish (10/11; 90.9%), and that they should be shared with the patient’s caregivers (11/11; 100%). GMH healthcare workers were split on the most useful format (link to CancerQuest = 36%; app = 27%; every format = 18%), and the barriers in sharing the videos (information overload = 18%; access/technology = 18%; patients are not tech‐savvy = 18%; no barrier = 18%; other barrier = 45%). The other barriers noted by health care workers included: finding the right person to disseminate the videos, patients actually using the videos, the lack of time in clinic as they have to see many patients in one day, and making the videos a part of the clinic process.

#### 3.1.3 Winship network sites

Four healthcare workers at Winship network sites participated in this study. The majority felt that it would be best for patients to view the videos before or during consent in the clinic (100%), either on a computer/TV/tablet (75%), or during the in‐clinic teaching (50%). Healthcare workers all agreed that the best person to introduce the videos would be a nurse (100%), that the videos were appropriate (100%), that they should be translated to Spanish (100%), and that they should be shared with the patient’s caregiver (100%). Network healthcare workers mentioned that a link to CancerQuest would be the most useful format (75%). Time constraints in the clinic were the most frequent barrier mentioned (75%), and healthcare workers suggested sending the patients home with information about the videos as well.

Table 2 outlines each site’s preferred dissemination method and the dissemination method used. Comparing the major similarities and differences between the three sites, Winship clinicians recommended that the videos should be viewed at home using technology rather than in the clinic (*p* = 0.0033) and that an app was the best format (*p* = 0.0081) significantly more often than Grady or Network participants. There were no significant differences among the three sites about who the healthcare workers thought should introduce the video, whether they were appropriate, if they should be translated, and if they should be shared with a patient’s caregiver (Table 3).

**TABLE 2:** Comparison of the interview results at each site to the implementation plan used

Site	Preferred dissemination methods	Dissemination method used
Winship	Smart application (68.2%)	1. Development of a smart application in the future2. Social media campaign since IT experts stated this was easier access than through portal
	Link through the patient portal (40.9%)	
	Handout with link to CancerQuest (27.2%)	
Grady	In clinic viewing (72%)	1. Computer browsers in patient rooms automatically open to the webpage2. Flyer with link to CancerQuest is placed in every new patient folder
	Using computer screens/ monitors (54%)	
Winship Network sites	In clinic viewing (100%)	1. Flyers sent to each clinic site2. Videos being utilized during in‐clinic patient education
	On a computer/TV/tablet (75%)	
	During the in‐clinic teaching (50%)	
	Using the CancerQuest link (75%)	

**TABLE 3:** Comparison of the major similarities and differences between providers suggestions of the best manner to disseminate the videos across the three different sites

	Winship Cancer Institute (*n* = 22)	Grady Memorial Hospital (*n* = 11)	Winship network sites (*n* = 4)	*p*‐value^a^
Videos should be viewed in the clinic	6 (27.2%)	8 (72%)	4 (100%)	**0.0033**
App in the best format to have the videos in	15 (68.2%)	3 (27%)	0 (0%)	**0.0081**
Nurse should introduce the videos	15 (68.2%)	9 (81.8%)	4 (100%)	0.5518
Videos are appropriate for patient population	10 (63.6%)	9 (81.8%)	4 (100%)	0.0411
Videos should be translated to Spanish	14 (63.6%)	10 (90.9%)	4 (100%)	0.1785
Videos should be shared with patient's caregiver	16 (72.7%)	11 (100%)	4 (100%)	0.0887

Bold values indicates, statistical significance was assessed at the 0.05 level.

^a^ *p*‐values are determined using Fisher’s exact tests.

### 3.2 Dissemination plans

#### 3.2.1 Winship Cancer Institute

**FIGURE 1:**
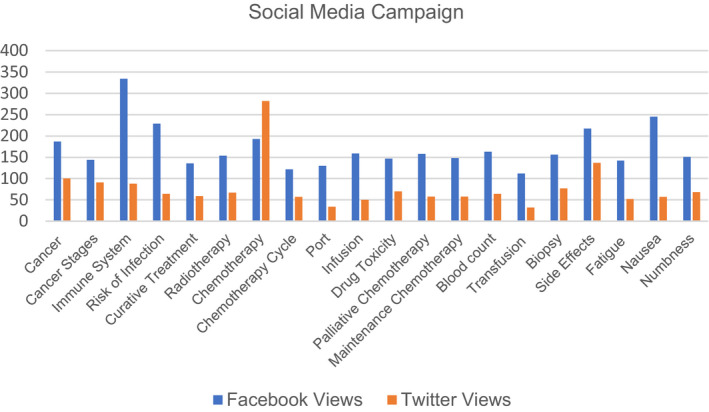
Results from the Winship social media campaign show total views since June 1, 2021. Videos were posted in the order listed (Cancer‐Numbness), with the first video posted on January 27, 2021, and the last video posted on March 19, 2021

Although the Winship interviewees preferred an app as the best dissemination method, due to the high production cost of developing a smart application, we have had to postpone the development of an app until additional funding is obtained. Winship interviewees' second‐most favored dissemination approach was to share the CancerQuest link via the patient portal, as their patient population tends to be more tech‐savvy. The research team consulted with a senior manager in web and digital initiatives who suggested that a social media campaign could be launched to make the videos readily available to patients without them having to log on to their personal patient portal. Given this advice, the health literacy videos were posted on Facebook and Twitter using the Winship social media page. The number of views was tracked and recorded. For every video except for “Cancer,” Facebook had more views than Twitter (Figure 1). Since sharing the CancerQuest link with patients was a preferred method, we also conducted an educational session about CancerQuest at a faculty meeting and encouraged its use.

#### 3.2.2 Grady Memorial Hospital

**FIGURE 2:**
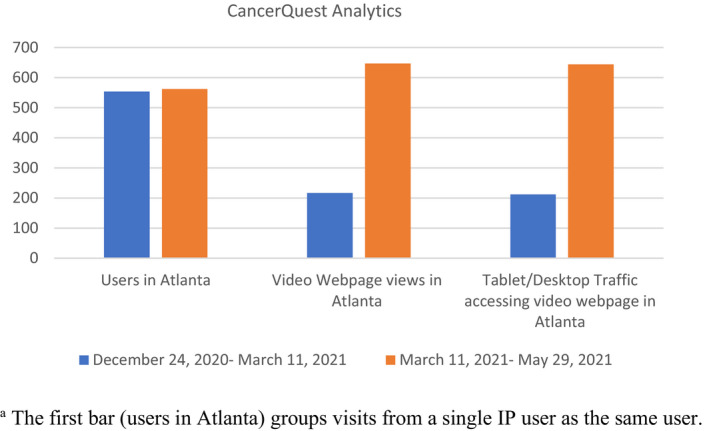
Results are from CancerQuest Analytics. Dissemination methods at GMH began starting March 11. The results show the views before and after dissemination. *Note*: ^a^The first bar (users in Atlanta) groups visit from a single IP user as the same user

Based on the results from the interviews with GMH healthcare workers, the majority of healthcare workers favored an approach that included the incorporation of videos within the clinic. Therefore, the ethics team consulted with a team of healthcare workers at GMH and developed five different tactics to incorporate the videos at GMH. One, in every patient room the computer browser opens automatically to the webpage with the videos. Two, there is a flyer put in every new patient folder and attached to discharge papers that has both the link and QR code to the videos. Three, the GMH webpage will be refreshed in 2021, and they are hoping to post the videos there. Four, there is going to be a system‐wide update at GMH, and they are hoping to add the videos to the discharge summary paperwork. Five, they plan to utilize the text reminder app WELL in July 2021 and hope to add the videos to this platform as well. Analytics from CancerQuest were used to track the preliminary results from this implementation plan. The clinic room’s browsers began showing the videos in each patient room starting on March 11, 2021. Since this date, the number of visits to the media center webpage in Atlanta and desktop traffic in Atlanta has significantly increased compared to the previous months (Figure 2).

#### 3.2.3 Winship network sites

**FIGURE 3:**
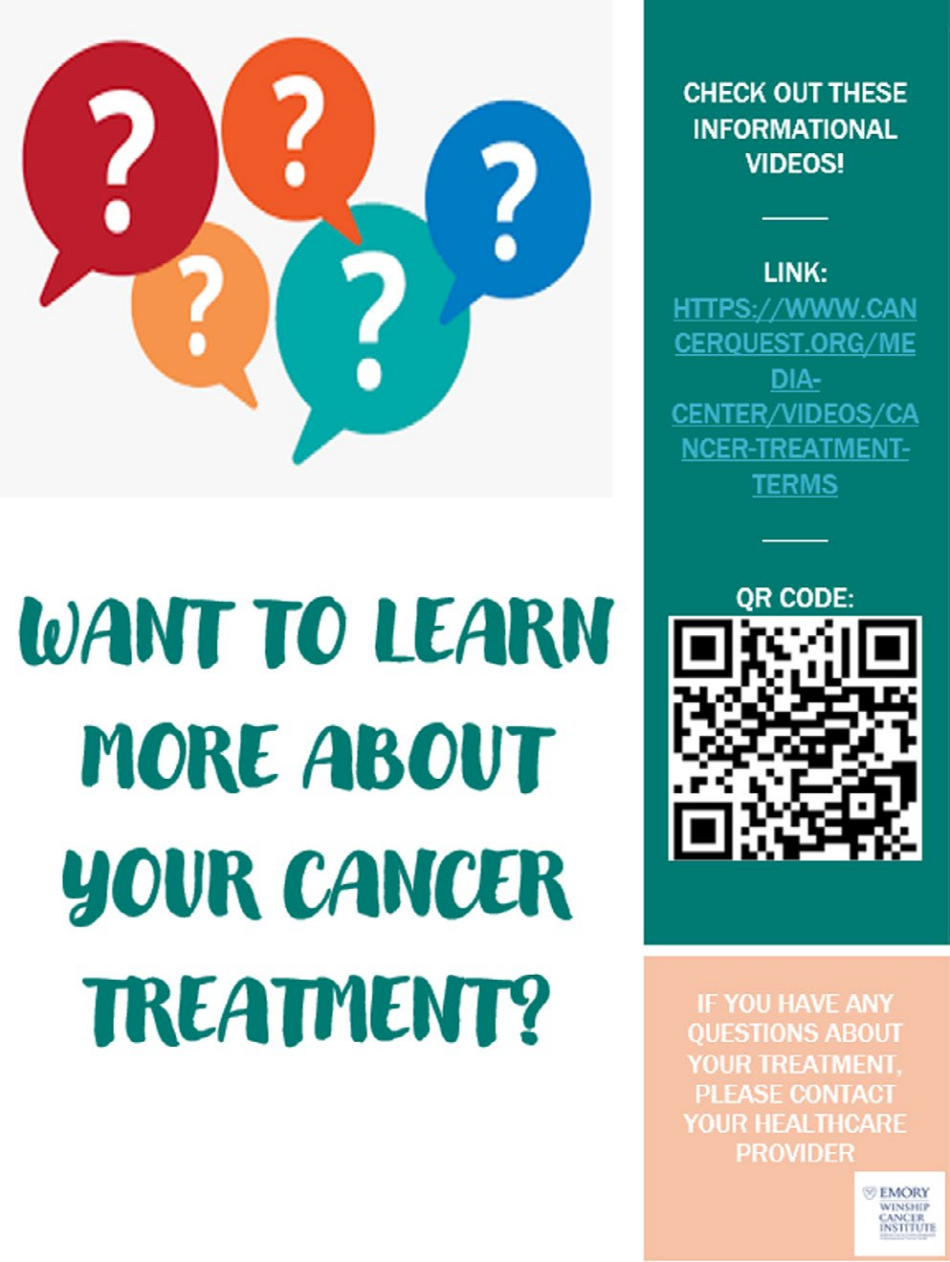
Sample flyer given to the Winship Network sites to incorporate into chemotherapy education

Since the majority of healthcare workers at the Winship network site favored an in‐clinic dissemination method as well, flyers that have both the QR code and link to the views have been sent to each clinic site. In addition, since incorporating the videos into the in‐clinic teaching was a suggested approach, the ethics team worked with key contacts in patient education at the network sites and they plan to utilize the videos during the in‐clinic patient education. A sample flyer that was sent to each site is shown in Figure 3. The ethics team held an update meeting with Winship Network coordinators in October 2021 to re‐share the fliers and encourage their use.

## 4 DISCUSSION

Disseminating educational materials is a crucial step in ensuring that the tools reach the patient populations they are supposed to serve, but often educational materials fail to be properly disseminated.^18^ Our results show that different clinic sites require different dissemination methods, depending on the patient population served. Winship, a higher socioeconomic status hospital, favored a more tech‐savvy approach, with the majority of clinicians stating that an app would be the best dissemination method (68.2%). In comparison, GMH, located just six miles from Winship, felt that it would be better for patients to view them in the clinic on a desktop monitor (75%), as they were more concerned that their patients may not have access at home. The Network sites shared a similar sentiment to the GMH healthcare workers and also had concerns about patients’ ability to access technology. However, they suggested a different approach than the GMH healthcare workers, stating that implementing the videos during the in‐clinic teaching would be the most helpful (50%), whereas the GMH healthcare workers wanted the videos to be displayed in patient rooms on the desktop monitor. These results highlight the importance of tailoring implementation measures of educational materials to each individual setting, which is congruent with the findings of past research on implementation science.^24^, ^25^

While healthcare workers tended to favor a particular method of dissemination, most healthcare workers suggested that it would be useful to have multiple different methods of dissemination, which past studies have shown to be an effective approach.^26^ Therefore, we implemented a variety of different dissemination methods at each clinic site. Since the Winship patient population was more tech‐savvy, we created a social media campaign, as social media has been an effective way to inform patients.^27^ As evident from Figure 1, the social media campaign helped increase the number of views of each video, indicating that social media is an effective way to share educational resources with patients, especially those who are more tech‐savvy. The social media campaign results do suggest that Facebook might be the most useful platform to share these educational resources, as it generated more views than Twitter and research has shown it to be the most popular social media platform among adults.^28^ At GMH, the significant increase in webpage access and desktop/tablet traffic in Atlanta indicates that the in‐clinic dissemination plan is working for that specific patient population. While this only showcases preliminary results, the increase in video views is encouraging. Although the dissemination methods at Winship and GMH are quite different, as one involves no clinic time, while the other is highly dependent on in‐clinic viewing, the success of both implementation measures further highlights the importance of tailoring the dissemination of educational materials to meet the needs of each specific patient population. While we have not been able to track the frequency of implementation of the videos during the in‐clinic teaching at Network sites, we are hopeful that this method of dissemination will work.

There were a few notable limitations to this study. Only a small number of Winship Network site clinicians participated in this study, so saturation of themes was not reached. More research with an increased number of participants should be conducted to determine the best dissemination tactics in rural clinics. Additionally, we interviewed a more diverse group of healthcare workers at the Grady site than at Winship and the Network sites and this difference may have influenced the differing results at each site. Also, while many Winship clinicians had suggested a smartphone application as the best method for dissemination, due to the high costs of producing an application, the application development will be a future project. It was also difficult to track the implementation measure at the network sites, so we were unable to provide any tangible results, such as the number of flyers that were distributed to patients. For future research, it would be useful to increase the number of healthcare workers interviewed about their preferred dissemination methods, particularly at rural sites, and to interview patients about the acceptability of the implementation methods and suggestions for other preferred methods.

## 5 CONCLUSION

Educational materials explaining oncology treatment terminology enhance patient‐centered care, yet without proper dissemination, these crucial educational tools may never reach the intended patient population. The healthcare settings examined in this study served significantly different patient populations and the dissemination recommendations were quite different. Healthcare workers serving lower SES patients believed that the underserved patients needed the videos accessible in the clinic whereas healthcare workers serving higher SES patients thought that a web‐based app, requiring a smart device, or internet access, was more appropriate.

Our study highlights the importance of utilizing site‐specific dissemination methods in order to ensure that all populations have access to educational tools, particularly those educational tools that rely on technology. Our preliminary dissemination tracking suggests that using a location‐specific dissemination plan may work to increase patient’s access to educational tools. Without a location‐specific dissemination plan, educational tools may only be available to those with means, thus further exacerbating disparities, rather than alleviating them.

## CONFLICT OF INTERESTS

The authors report no conflict of interests.

## ETHICS STATEMENT

The research protocol was approved by the Emory IRB. All subjects provided verbal consent before the interview and consent was documented by completion of the interview.

## AUTHOR CONTRIBUTIONS

Shannon Blee‐Investigation, Writing Original Draft; Jamil Facdol, Investigation, Writing‐review and editing; Margie Dixon‐ Conceptualization, Data Curation, Methodology, Project Administrator, Writing‐review and editing; Viraj Master‐ Conceptualization, Writing‐review and editing; Jeffrey Switchenko‐ Formal analysis, Methodology, Writing‐review and editing; Rebecca D. Pentz‐ Conceptualization, Methodology, Supervision, Writing‐review and editing.

## Data Availability

The data that support the findings of this study are available from the corresponding author upon reasonable request.
